# The influence of general practitioner and patient sex on the treatment of major depression

**DOI:** 10.3389/fphar.2023.1274774

**Published:** 2023-11-09

**Authors:** Elisabeth Jufresa-Blanch, Neus Carrilero, Anna García-Altés

**Affiliations:** ^1^ Department of Medicine and Life Sciences, Universitat Pompeu Fabra, Barcelona, Spain; ^2^ Agència de Qualitat i Avaluació Sanitàries de Catalunya (AQuAS), Barcelona, Spain; ^3^ Department of Experimental and Health Sciences (DCEXS), Universitat Pompeu Fabra, Barcelona, Spain; ^4^ Institut de Recerca de l’Hospital de la Santa Creu i Sant Pau, Barcelona, Spain; ^5^ CIBER de Epidemiología y Salud Pública (CIBERESP), Barcelona, Spain; ^6^ Institut d’Investigació Biomèdica (IIB Sant Pau), Barcelona, Spain

**Keywords:** antidepressive agents, sexism, primary healthcare, general practitioners, major depressive disorder

## Abstract

**Introduction:** Biological and sociocultural factors may lead to a significant gender bias in the treatment of major depression and thus contribute to accentuating gender inequalities. However, the influence of the general practitioner’s (GP’s) sex on the prescription of antidepressants has not been adequately assessed in previous work and remains unclear. This retrospective cohort study aims to determine the influence of GP and patient sex on the treatment of major depression.

**Methods:** The study population comprised 87,629 patients (33.56% male patients and 66.44% female patients) aged over 15 years newly diagnosed with major depression recorded between 2017 and 2019 in Catalonia, Spain. Logistic regression models were used to evaluate the effect of GP sex on the therapeutic strategy (i.e., whether antidepressants were prescribed at the first diagnostic visit). Cox proportional hazards models and survival analyses were conducted to compare, according to GP and patient sex, the probability that a patient would be prescribed an antidepressant at any time during the study period. Finally, a multiple linear regression analysis was performed to assess the pharmacological intensity of the treatment [monthly fluoxetine-equivalent defined daily dose (DDD)].

**Results:** Female patients were more likely to be prescribed an antidepressant at the time of diagnosis, both by male [OR = 1.11, 95% CI = (1.05, 1.17), *p* < 0.001] and female GPs [OR = 1.13, 95% CI = (1.09, 1.17), *p* < 0.001]. Similarly, female patients were 8% and 9% more likely than male patients to be prescribed an antidepressant from male [HR = 1.08, 95% CI = (1.05, 1.11), *p* < 0.001] and female GPs [HR = 1.09, 95% CI = (0.92, 1.07), *p* < 0.001], respectively, during the study period. Female GPs prescribed less antidepressants than male GPs: an average of 0.39 less monthly fluoxetine-equivalent DDD [*β* = −0.39, 95% CI = (0.10, −3.92), *p* < 0.001].

**Discussion:** Few differences are observed between male and female GPs regarding the therapeutic strategy and its intensity for the treatment of major depression. However, both male and female GPs are influenced by biases and stereotypes that entail differential antidepressant-prescribing behaviors in accordance with the sex of the patient and their characteristics.

## Introduction

Major depression is a common mental health disorder that severely impacts psychosocial functioning and impairs the quality of life (Malhi and Mann, 2018). It is estimated that approximately 280 million people worldwide (3.8% of the world population: 3.0% of men and 4.5% of women in 2019) of all ages and social conditions suffer from depressive disorders (Institute for Health Metrics and Evaluation, 2021). Over the course of a lifetime, major depression affects women twice as frequent as men, with a peak in prevalence in the second and third decades of life for both genders (Malhi and Mann, 2018) and a higher prevalence among those who are most socially and economically deprived (World Health Organization. Regional Office for Europe, 2015).

Depression is a complex multifactorial process (Chiriţă et al., 2015), and its probability of development depends on a broad group of risk factors to which people are exposed over their lifetime. Adverse experiences in childhood (e.g., school bullying, abuse, or domestic violence) (Fryers and Brugha, 2013; Jensen et al., 2013), disadvantaged social situations (e.g., job insecurity, inadequate social network, or low income), or setback events (e.g., grief, loss of status, or physical health problems) play an influential role in exacerbating the progression of depression in adulthood (Ajuntament de Barcelona, 2018; Remes et al., 2021).

According to the Health Survey of Catalonia (Enquesta de salut de Catalunya, ESCA) in 2021, 9% of the Catalan population aged over 15 years were reported to be suffering from major depression or severe major depression, with a higher prevalence in women (12.2%) than in men (5.7%). The rate increased with age, especially in the age group of over 75 years (13.1% compared to 7.4% in the age group of 15–44 years), and was higher in people from the most vulnerable social class (10.8% compared to 4.8% in the wealthiest) and with a lower educational level (14.6% in those with primary schooling or lower compared with 5.5% in those with university education) (Schiaffino and Medina-Bustos, 2022). These data highlight the intersectionality between the multiple social identities that favor the perpetuation of structural health inequalities (Bowleg, 2012). Regarding drug consumption, it is estimated than 16% of men and 25% of women in Catalonia had consumed at least one psychotropic drug in 2018 (Observatori del Sistema de Salut de Catalunya, 2018).

Depression in adults must be treated in accordance with a stepped care model in collaboration between primary care and mental health centers (National Institute for Health and Care Excellence, 2022). Primary care centers act as the first contact of patients within the healthcare system (Kealy et al., 2019). It is estimated that approximately 60% of mental healthcare is provided in the primary care setting, and 79% of antidepressant prescriptions are made by GPs (Park L and Zarate C, 2019). Thus, GPs play a fundamental role in the diagnosis, treatment, and follow-up of depression (Aragonés-Benaiges et al., 2014). The guidelines for treating depression (National Institute for Health and Care Excellence, 2022) emphasize that an observation period should be established to individually determine the best treatment and that other psychological and behavioral interventions should be prioritized before, or at the same time as, pharmacological treatment. Great care is taken to avoid the administration of drugs that might lower the mood and exacerbate depression, to attend to any substance abuse, and to promote healthy lifestyle habits such as smoking cessation, sleep hygiene, regular exercise, and a balanced diet (Malhi and Mann, 2018).

Another important aspect of any health treatment (depression being no exception) is the therapeutic strategy proposed for each patient. This choice may be biased by socioeconomic or sex characteristics. Previous research on gender bias focuses on variables such as the different types of therapeutic strategies, consumption, and expenditure on medicines by sex (Ruiz-Cantero and Verdú-Delgado, 2004). In this regard, the evidence suggests that emotional distress in female patients is often medicalized and that there is a tendency among health professionals to attribute physical symptoms to psychological factors more readily in female patients than in male patients, instead of looking for other attributable biological causes of their symptoms (Valls-Llobet, 2020).

Following on from this, several studies in areas other than mental health have shown that GP characteristics may contribute to important differences in medical attention, particularly in terms of prescribing medication and quality of care. Compared to their male peers, female GPs were shown to adhere more closely to clinical guidelines, dedicate more time to their patients, and provide more patient-centered care (Mishra et al., 2020). These differences in attitudes toward prescribing between GP sexes highlight the importance of beliefs associated with gender, both in doctor–patient relationships and in drug prescriptions (Gil García et al., 2005). More specifically, regarding psychotropic drugs, the evidence suggests that doctor–patient relationships are influenced by pre-existing gender stereotypes and biases toward patients, with women being perceived as more emotionally sensitive and men being perceived as more vigorous. Consequently, male GPs tend to prescribe more psychotropics to female patients (McIntyre et al., 2021). Other studies, however, have not found differences between GP sexes, with both demonstrating significant biases to the detriment of female patients (Morabia et al., 1992).

Although stereotypes and gender biases toward patients are known to influence prescribing behavior (McIntyre et al., 2021), the morbidity, social and economic impact, and inadequate treatment associated with major depression underline the need to investigate in greater depth the variation in antidepressant prescription according to sex and its implications for clinical outcomes. In this way, this study aims to analyze the treatment of major depression with antidepressant drugs and to determine the influence of sex of both the GP and the patient on the likelihood of being prescribed an antidepressant drug and thus establish possible improvements in the Catalan public health system and identify optimal prescribing practices for mental health disorders. This research is part of the strategic line proposed in the Catalan Health Plan 2022–2025 to guide the health system toward community care practices and non-pharmacological interventions to address situations of emotional and social distress (Generalitat de Catalunya. Departament de Salut, 2021).

## Materials and methods

### Design and study population

This study is a population-based retrospective cohort study comprising individuals aged 15 years and above newly diagnosed with major depression between 2017 and 2019, as recorded in the primary care public health system of Catalonia, Spain.

### Data collection

Healthcare in Catalonia is organized as a National Health System, funded by taxes. All of Catalonia’s residents (7,348,275 as of 2017) are granted universal public healthcare coverage by law (Servei Català de la Salut, 2017). The use of publicly funded healthcare services is free, including primary care, specialized care, psychiatric care and mental health, urgent care, and social and healthcare, among others (Servei Català de la Salut, 2021). The sole exception is drug prescription, which is based on a co-payment system (Servei Català de la Salut, 2022). Each resident is assigned a personal healthcare ID which can be used to trace their use of healthcare services (Servei Català de la Salut, 2023b).

In this study, three different databases were used: the registry of insured persons (Registre central d’assegurats, RCA) (Servei Català de la Salut, 2023a) was used to obtain the reference population for the period 2017–2019. This registry compiles sociodemographic and socioeconomic information based on annual income level, employment status, and social security benefits of each individual, which are routinely used to calculate their pharmaceutical co-payment levels.

The minimum basic dataset of primary care (conjunt mínim bàsic de dades d’atenció primària, CMBD-AP) is an administrative registry that includes detailed information about sociodemographic characteristics and medical diagnoses [coded using the International Classification of Diseases (ICD), 10th Edition] drawn up from the information provided by all health centers in Catalonia. The CMBD-AP must be notified regarding all the activity of primary care, which encodes all contacts with primary care centers in the public health system at an individual level (Servei Català de la Salut, 2023a), recording, among other clinical care variables, the health problem reason for the medical visit and the date when the pathology was first diagnosed (Amado et al., 2018). The present study selected all patients first diagnosed between 2017 and 2019 with any codes under the categories F32 and F33 of the ICD-10-ES (Fourth Edition 2022), corresponding to major depressive disorders.

The electronic prescription database (sistema d’informació de la recepta electrònica, SIRE) (Generalitat de Catalunya, 2014) is an administrative database providing data on outpatient dispensing by the public health system at primary care centers. From this database, we obtained all the doses prescribed in DDDs, the Anatomical Therapeutic Chemical (ATC) code, the date, and the sex of the prescriber for each antidepressant prescription.

### Variables

#### Dependent variables

The main outcome variables were related to the pharmacological treatment of major depression and its therapeutic strategy, i.e., whether an antidepressant was prescribed simultaneously with the diagnosis, the time to prescription between the diagnosis and the antidepressant prescription, and the pharmacological intensity of the treatment.

We assessed the therapeutic strategy as a dichotomous variable by identifying patients who were receiving an antidepressant prescription at the time of their diagnosis. From the combination of the three datasets, we determined whether the patient’s first antidepressant prescription [according to the ATC code N06A (WHO Collaborating Centre for Drug Statistics, 2022)] coincided with the date of diagnosis of major depression. We excluded patients treated with antidepressants during the 4 months prior to the diagnosis of major depression to ensure that the pathology/treatment events were new and indicated for the depressive disorder.

The time to prescription was defined as the time interval between the diagnosis of major depression and the first antidepressant prescription. To assess this variable, we constructed a cohort, setting time 0 for each individual to the time of their diagnosis.

To compare the pharmacological intensity of antidepressant treatment, determined as the therapeutic potency of the antidepressant drug as a function of dose and dosing interval, we converted the DDD of all antidepressants prescribed for each month into monthly fluoxetine-equivalent DDD (see Supplementary Table 1). We excluded tianeptine prescriptions (which accounted for 0.06% of the total) due to a lack of fluoxetine-equivalent DDD data.

#### Independent variables

The independent variables were related to the characteristics of patients (sex, age, nationality, socioeconomic position (SEP), and adjusted morbidity groups [Grups de morbiditat ajustats, GMA]) (Vela et al., 2021) and their GP (sex).

The sex of the GP was treated as a dichotomous variable (male or female). Given that the GP who made the diagnosis may not have been the one that made the prescription, we recorded data from the first prescribing GP. If there was no prescription, the GP’s sex was considered to be that of the diagnosing GP. We considered this approach because not prescribing antidepressants is also a therapeutic decision. For the intensity of the treatment outcome variable, we used the prescribing GP’s sex for each monthly antidepressant prescription.

Patient characteristics include sex as a dichotomous variable (male or female), age categorized into five groups (15–24 years, 25–44 years, 45–64 years, 65–74 years, and 75 years and older), nationality (Spanish or other), SEP, and GMA score. SEP was proxied by patients’ pharmaceutical co-payments obtained from the RCA registry. The pharmaceutical co-payment system uses income level and employment status information to define three categories: “medium–high” (≥18,000 €/year), “low” ( < 18,000 €/year), and “very low” (no member of the household is employed or receiving welfare support). GMA is a morbidity index that determines the individual’s health risk using health administrative data compiled in the Catalan Health System (Vela et al., 2021).

### Statistical analysis

To carry out the statistical analysis, we constructed two databases. The first one included all patients newly diagnosed with major depression between 2017 and 2019 and their first antidepressant prescription, if any. From these data, we assessed the “therapeutic strategy” and “the time to prescription” for each patient. The second database included only the patients with prescriptions, and it was used to determine the intensity of the treatment.

First, a descriptive analysis was conducted to determine differences between the patient’s sex, the GP’s sex, and the dependent and independent variables. Proportion tests and chi-squared tests (for categorical variables) and t-tests or Mann–Whitney U-tests (for continuous variables) were conducted, depending on the variable.

We then performed a logistic regression analysis to determine differences between the GPs’ sexes in terms of the therapeutic strategy (i.e., whether antidepressants were prescribed on the diagnostic visit or not). Three models were created: one for each sex of the prescribing GP and a third one introducing the sex of the prescribing GP as an explanatory variable. We obtained ORs for the different independent variables with their 95% CIs.

Afterward, Cox proportional hazards models were conducted to assess the outcome of the time to prescription, determining the effect of the characteristics (sex, age, and SEP) of a patient newly diagnosed with major depression on the time he or she would be prescribed an antidepressant drug during the analysis period (3 years maximum). This model was computed for both GP sexes. Moreover, we also conducted a survival analysis using the Kaplan–Meier method and the Gehan–Breslow (generalized Wilcoxon) test to describe the observational period according to patient characteristics and GPs’ sex.

Finally, for the continuous dependent variable, monthly fluoxetine-equivalent DDD, a multiple linear regression analysis was performed. The variables and the stratification strategy were the same as in the logistic regression analysis mentioned previously. The *β* coefficients were obtained for the different independent variables accompanied by their 95% CIs.

All computed models were adjusted for the following patient characteristics: age, nationality, SEP, and GMA.

All statistical analyses were carried out using R version 4.1.2 software (The R Foundation, 2002). *p*-values below 0.05 were considered statistically significant.

## Results

The total study population comprised 87,629 patients (33.56% male patients and 66.44% female patients) newly diagnosed with major depression between 2017 and 2019; 62.13% of these patients (35.06% male patients and 64.94% female patients) aged between 25 and 64 years, and 19.26% (22.62% male patients and 77.38% female patients) had a very low SEP. In all, 29,434 patients (35.17% male patients and 64.83% female patients) were treated by a male GP, while 56,566 patients (32.69% male patients and 67.31% female patients) were treated by a female GP, and in 1,629 patients (1.86% of all patients), the sex could not be determined. The characteristics of the patients according to the GP’s sex are given in Table 1.

**TABLE 1:** Characteristics of patients newly diagnosed with major depression by their GP’s sex for the period of 2017–2019 recorded in Catalonia, Spain.

	Male general practitioner			Female general practitioner		
Variable	Male patients N (%)	Female patients N (%)	*p*-value	Male patients N (%)	Female patients N (%)	*p*-value
Age (years)						
15–24	616 (6.00%)	989 (5.20%)	<0.001^a^	1, 089 (5.90%)	2, 046 (5.40%)	<0.001^a^
25–44	2, 877 (27.80%)	4, 942 (25.90%)	<0.001^a^	4, 918 (26.60%)	9, 534 (25.00%)	<0.001^a^
45–64	3, 903 (37.70%)	6, 784 (35.60%)	<0.001^a^	7, 005 (37.90%)	13, 487 (35.40%)	<0.001^a^
65–74	1, 213 (11.70%)	2, 447 (12.80%)	<0.001^a^	2, 118 (11.50%)	5, 050 (13.30%)	<0.001^a^
≥75	1, 743 (16.80%)	3, 920 (20.50%)	<0.001^a^	3, 364 (18.20%)	7, 955 (20.90%)	<0.001^a^
Nationality						
Spanish	6, 364 (61.50%)	11, 854 (62.10%)	0.280^a^	11, 367 (61.50%)	23.728 (62, 30%)	0.047^a^
Other	3, 988 (38.50%)	7, 271 (37.90%)	0.280^a^	7, 127 (38.50%)	14, 344 (37.70%)	0.047^a^
Socioeconomic position						
Medium–high	3, 516 (34.00%)	4, 820 (25.30%)	<0.001^a^	6, 433 (34.80%)	9, 822 (25.80%)	<0.001^a^
Low	5, 465 (52.80%)	9, 988 (52.40%)	<0.001^a^	9, 685 (52.40%)	19, 640 (51.60%)	<0.001^a^
Very low	1, 365 (13.20%)	4, 267 (22.40%)	<0.001^a^	2, 366 (12.80%)	8, 590 (22.60%)	<0.001^a^
GMA score						
Median, first to third quantile	8.83 [4.20, 16.76]	8.91 [4.54, 16.24]	0.156^b^	9.28 [4.41, 18.90]	9.31 [4.68, 16.82]	0.001^b^
Antidepressant prescription						
Yes	5, 539 (53.50%)	10, 909 (57.20%)	<0.001^a^	9, 257 (50.10%)	20, 497 (53.80%)	<0.001^a^
No	4, 813 (46.50%)	8, 173 (42.80%)	<0.001^a^	9, 237 (49.90%)	17, 575 (46.20%)	<0.001^a^
Fluoxetine-equivalent DDD						
Median, first to third quantile	46.62 [30.60, 65.14]	45.50 [30.60, 62.16]	<0.001^b^	43.80 [30.60, 62.16]	45.08 [30.60, 62.16]	<0.001^b^

Note:

a Chi-squared test.

b Mann–Whitney U-test.

DDD, defined daily dose; GMA, adjusted morbidity groups (Grups de morbiditat ajustats); GP, general practitioner.

### Therapeutic strategy: antidepressant prescription at the time of diagnosis

**FIGURE 1:**
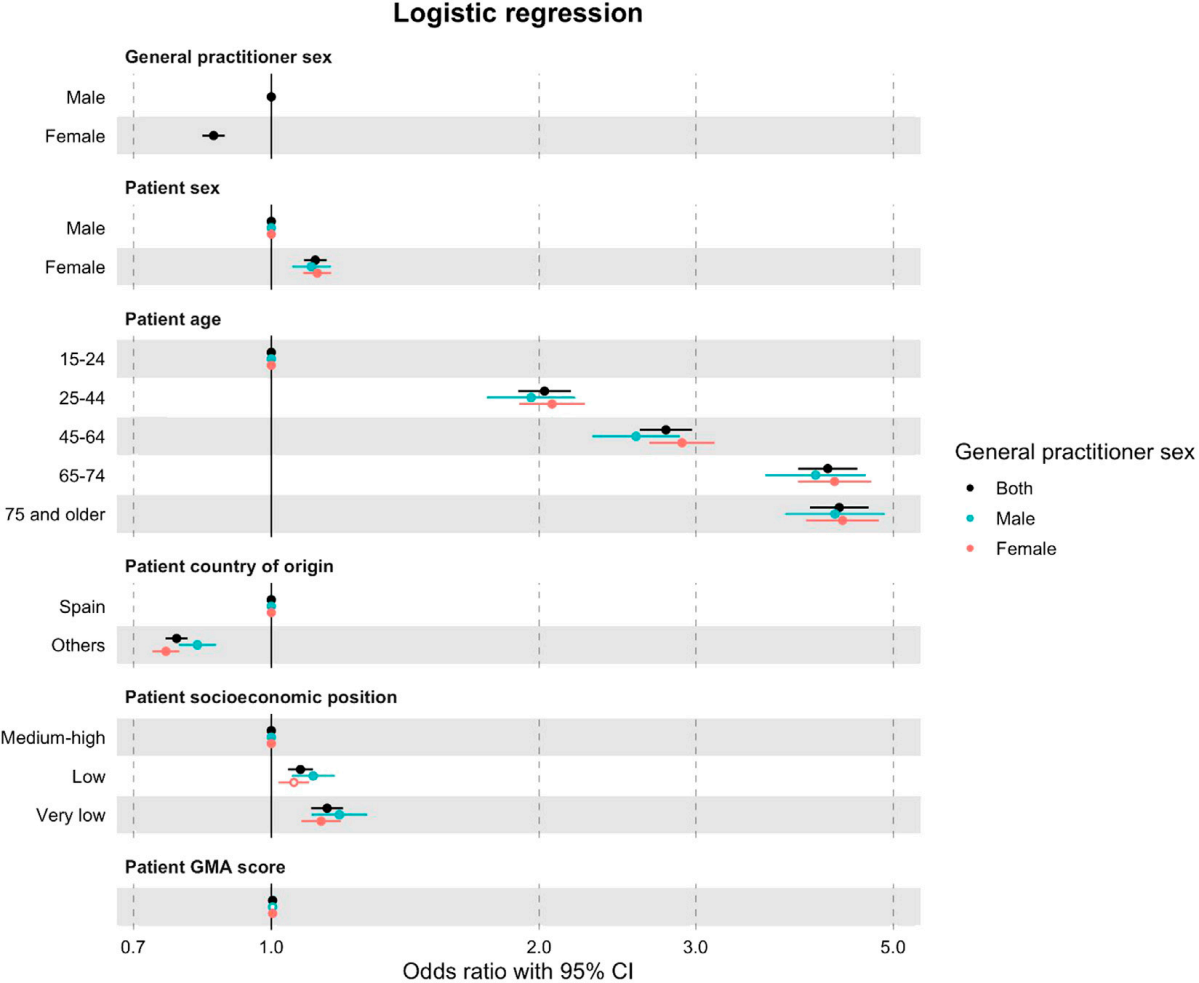
Logistic regression analyses (95% CI) with OR of patients prescribed/not prescribed an antidepressant at the time of their diagnosis of major depression, according to patient characteristics and the sex of their GP for the period of 2017–2019 recorded in Catalonia, Spain. Regression models were adjusted for patient characteristics: age, nationality, SEP, and GMA. Reference categories: GP sex (male), patient sex (male), patient age (15–24 years), patient nationality (Spanish), and patient socioeconomic position (medium–high).

According to the logistic analysis (see Figure 1), female GPs prescribed 14% fewer antidepressants than their male counterparts [OR = 0.86, 95% CI = (0.84, 0.89), *p* < 0.001] at the time of diagnosis. Regarding patient’s sex, female patients had more probabilities of receiving an antidepressant at the time of diagnosis than male patients, from both male [OR = 1.11, 95% CI = (1.05, 1.17), *p* < 0.001] and female GPs [OR = 1.13, 95% CI = (1.09, 1.17), *p* < 0.001]. Regarding the patients’ age, the older the patient, the greater the chance of antidepressants being prescribed at the time of diagnosis by either a male or female GP. The same occurred with SEP, where the most vulnerable patients were more likely to receive a prescription than wealthier patients (particularly from male GPs who issued 5% more prescriptions to these patients than female GPs).

### Time to prescription between the diagnosis and the antidepressant prescription

**FIGURE 2:**
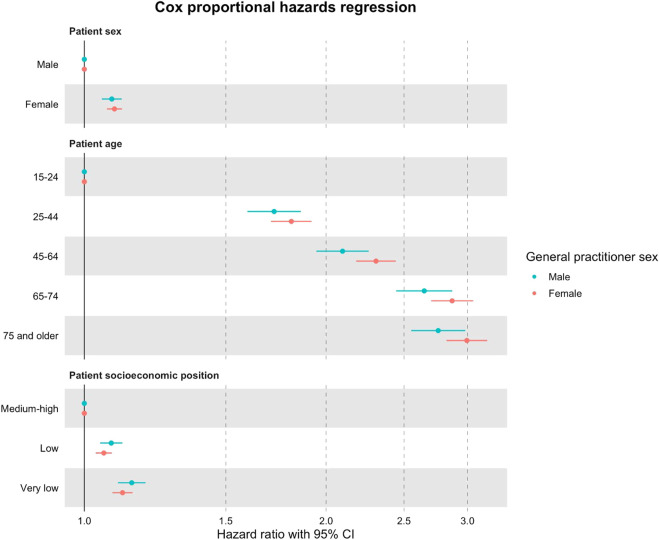
Cox proportional hazards models (95% CI) with HR of a patient prescribed an antidepressant drug during the study period after the major depression diagnosis, according to the patient’s characteristics and the sex of their GP for the period of 2017–2019 recorded in Catalonia, Spain. Regression models were adjusted for patient characteristics: age, nationality, SEP, and GMA. Reference categories: patient sex (male), patient age (15–24 years), and patient socioeconomic position (medium–high).

Figure 2 shows the Cox model of the time until an antidepressant prescription is issued. During the study period, female patients were 8% more likely than male patients to receive an antidepressant prescription from male GPs [HR = 1.08, 95% CI = (1.05, 1.11), *p* < 0.001] and 9% from female GPs [HR = 1.09, 95% CI = (0.92, 1.07), *p* < 0.001]. Gradients of age and socioeconomic position were also detected: older patients were 1.76 times more likely to be prescribed an antidepressant drug (by male GPs) and twice as likely (by female GPs) as younger people during the study period, and vulnerable patients were 15% (when treated by a male GP) and 12% (when treated by a female GP) more likely to be prescribed an antidepressant.

**FIGURE 3:**
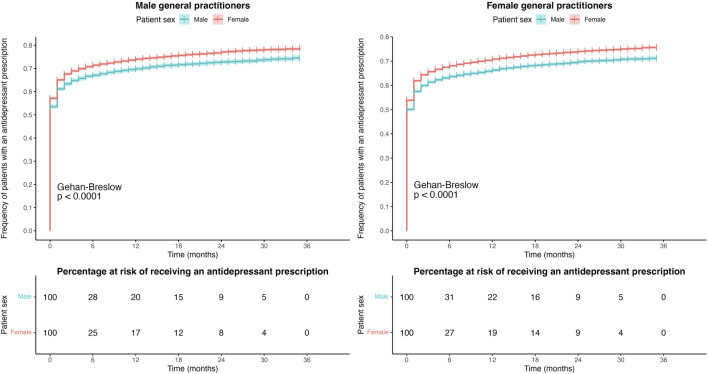
Kaplan–Meier curves for the time to prescription between the major depression diagnosis and the first antidepressant prescription according to patient sex for male and female GPs.

Figure 3 shows the Kaplan–Meier survival curve according to the patient’s sex for male and female GPs. Female patients treated by both male and female GPs received an antidepressant prescription sooner after diagnosis than male patients. In the case of male GPs, 54% of male patients and 57% of female patients were prescribed an antidepressant at the time of diagnosis compared with only 50% of male patients and 54% of female patients in the case of female GPs. One year after the diagnosis, 70% of male patients and 74% of female patients treated by male GPs had been prescribed an antidepressant drug, whereas for female GPs, these figures were 67% for male patients and 71% for female patients. At the end of the study period, 25% and 29% of male patients treated by male and female GPs and 21% and 24% of female patients treated by male and female GPs, respectively, had not received an antidepressant prescription yet.

**FIGURE 4:**
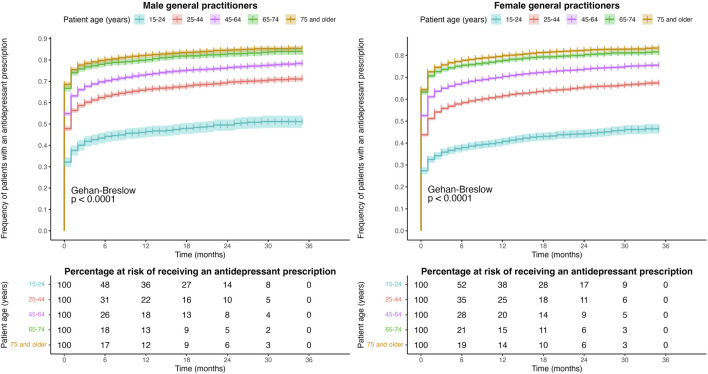
Kaplan–Meier curves for the time to prescription between the major depression diagnosis and the first antidepressant prescription according to patient age for male and female GPs.

**FIGURE 5:**
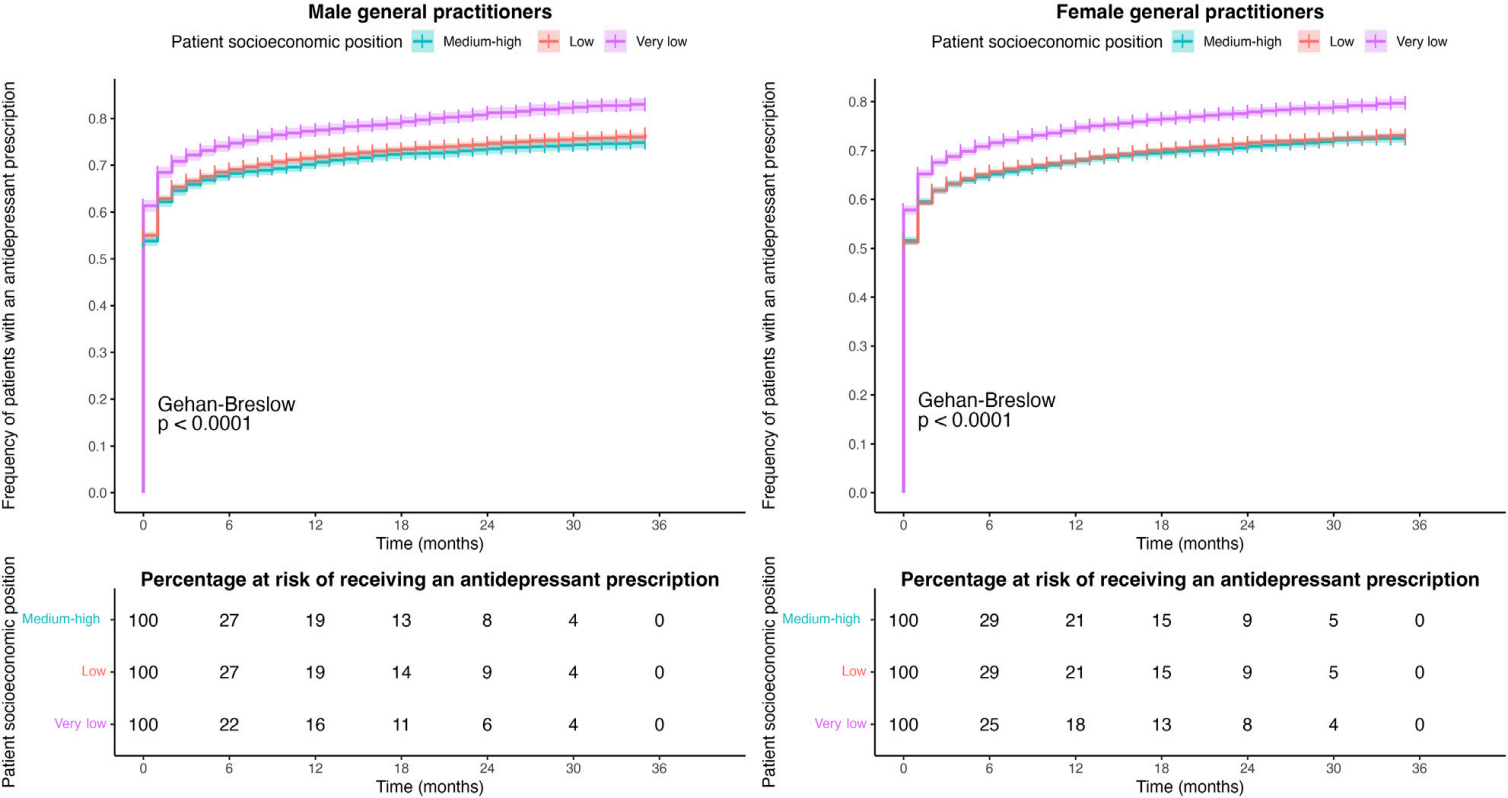
Kaplan–Meier curves for the time to prescription between the major depression diagnosis and the first antidepressant prescription according to the patient socioeconomic position for male and female GPs.

Figures 4, 5 show the Kaplan–Meier survival curve of patients according to their age and SEP, respectively, for male and female GPs. Both male and female GPs prescribed antidepressants at the time of diagnosis to a higher number of elderly patients than to the youngest patients, with rates of 69% vs. 32% and 64% vs. 27%, respectively. Similar results were observed with patients with “very low” and “medium–high” SEP, at 61% vs. 54% and 58% vs. 52%, respectively.

### Intensity of treatment

Female GPs prescribed an average of 0.39 less monthly fluoxetine-equivalent DDD than male GPs [*β* = −0.39, 95% CI = (0.10, −3.92), *p* < 0.001] (see Table 2). Male GPs prescribed an average of approximately 1 less monthly fluoxetine-equivalent DDD to female patients than to male patients [*β* = −1.03, 95% CI = (−1.37, −0.68), *p* < 0.001], while female GPs prescribed an average of 0.24 less monthly fluoxetine-equivalent DDD [*β* = −0.24, 95% CI = (−0.51, 0.20), *p* = 0.050]. In addition, in patients treated by male GPs, the age group of 45–64 years had the highest intensity of treatment, while in patients treated by female GPs, the age group of 25–44 years had the highest intensity of treatment. The maximum difference between monthly fluoxetine-equivalent DDD prescribed in older and younger patients was observed in female GPs (difference in DDD of 4.78 in male GPs and one of 7.67 in female GPs). Regarding the SEP, no notable differences were found according to the GP’s sex. Patients with the lowest SEP received more intense treatment than those with the highest SEP from both male and female GPs, although the results were significant in male but not in female GPs.

**TABLE 2:** Multiple linear regression (95% CI) with *β* coefficients of monthly fluoxetine-equivalent DDD prescribed to patients newly diagnosed with major depression according to patient characteristics and their GP’s sex for the period of 2017–2019 recorded in Catalonia, Spain.

	All general practitioners			Male general practitioners			Female general practitioners		
Variable	*β*	95% CI	*p*-value	*β*	95% CI	*p*-value	*β*	95% CI	*p*-value
General practitioner									
Sex									
Male	1.00			−	−	−	−	−	−
Female	−0.39	[0.10, −3.92]	<0.001	−	−	−	−	−	−
Patient									
Sex									
Male	1.00								
Female	−0.53	[0.11, −4.97]	<0.001	−1.03	[−1.37, −0.68]	<0.001	−0.24	[−0.51, 0.2]	0.050
Age (years)									
5–24	1.00			1.00			1.00		
25–44	2.09	[1.27, 2.90]	<0.001	1.80	[0.48, 3.12]	0.001	2.24	[1.21, 3.26]	<0.001
45–64	2.39	[1.60, 3.18]	<0.001	3.44	[2.16, 4.73]	<0.001	1.80	[0.81, 2.80]	<0.001
65–74	−2.55	[−3.36, −1.74]	<0.001	−1.14	[−2.47, −0.18]	0.050	−3.30	[−4.32, −2.27]	<0.001
≥ 75	−9.31	[−10.12, −8.51]	<0.001	−8.22	[−9.53, −6.90]	<0.001	−9.91	[−10.93, −8.89]	<0.001
Nationality									
Spanish	1.00			1.00			1.00		
Other	−1.86	[−2.07, −1.67]	<0.001	−1.85	[−2.18, −1.52]	<0.001	−1.88	[−2.13, −1.63]	<0.001
Socioeconomic position									
Medium–high	1.00			1.00			1.00		
Low	−0.76	[−0.98, −0.53]	<0.001	−0.11	[−0.49, −0.27]	0.100	−1.10	[−1.38, −0.82]	<0.001
Very low	1.03	[0.76, 1.29]	<0.001	2.20	[1.76, 2.65]	<0.001	0.39	[0.06, 0.72]	0.010
GMA score	0.16	[0.15, 0.16]	<0.001	0.11	[0.10, 0.13]	<0.001	0.18	[0.17, 0.19]	<0.001

Note: Regression models were adjusted for patient characteristics: age, nationality, SEP, and GMA.

CI, confidence interval; DDD, defined daily dose; GMA, adjusted morbidity groups (Grups de morbiditat ajustats); GP, general practitioner.

## Discussion

In general, few differences are observed between male and female GPs with regard to the pharmacological strategy and the intensity of the treatment of major depression. Both male and female GPs were prescribing antidepressants to more than 50% of their patients at the time of diagnosis of major depression. Female GPs tended to prescribe less intense treatments than their counterparts, although these differences do not lead to major variations in clinical practice. However, the sex of the patients and their characteristics had an influence on the treatment of this mental health disorder since it was found that female, elderly, and socioeconomically vulnerable patients were the most likely to receive an antidepressant prescription at the time of diagnosis by both male and female GPs.

The sex of the GP is perceived as a relevant factor in healthcare practice (Champagne-Langabeer and Hedges, 2021; Bouissiere et al., 2022), and our research has outlined several behavioral patterns that differ between male and female GPs. Compared to their counterparts, female GPs opt for pharmacological treatments of lower intensity and, in general, do not immediately make prescriptions on the first care visit. It is reported that female GPs spend more time with their patients and prescribe drugs more conservatively, tending to start with lower doses to avoid adverse effects and then adjust to clinical recommendations. Male GPs, on the other hand, tend to prescribe more invasive procedures for certain diagnoses (Mishra et al., 2020; McIntyre et al., 2021). However, these findings are not consistent enough to determine that the differences in a therapeutic strategy for major depression are attributable to the GP’s sex.

In our study, female patients treated by both male and female GPs were more likely to be prescribed antidepressant drugs at the time of their major depression diagnosis. The time to prescription was also shorter in female patients. During the observation period, GPs often schedule visits with mental health specialists in order to obtain a more accurate diagnosis. In this regard, results reported by the Catalan Healthcare System (Garciá-Altés et al., 2018) show that although mental health centers are frequented more often by female patients than male patients (3.0% vs. 2.4%), differences in the burden of mental health disease are more disparate (12.2% in female patients and 5.7% in male patients) (Schiaffino and Medina-Bustos, 2022). Therefore, we cannot consider equality in access to mental healthcare (European Institute for Gender Equality, 2021) if it seems that female patients have more unmet mental healthcare needs than male patients. These differences are even more marked for socioeconomic level and age.

To some extent, the sex gap recorded in the time to prescription may be due to the differences in patients’ patterns of reporting depression symptoms, that is to say, male patients are more likely to report more severe and acute symptoms (Shi et al., 2021) and also seek help from healthcare services later (Doblytė, 2020) due to the effects of social roles and the traditional conceptions of masculinity (Bambra et al., 2021). This notion is borne out by our results since the pharmacological treatment provided to male patients tended to be more intense. For their part, women may be subjected to the dual burden of employment and caring, which may entail higher rates of poverty, lower rates of education, and discrimination in the labor market, a circumstance that increases levels of stress, sadness, isolation, and uncertainty (Norman, 2004; Bambra et al., 2021). This situation may lead to poor mental health in women and higher levels of reporting and diagnosing mental health disorders and prescribing antidepressants than in men (Bacigalupe et al., 2020; Bacigalupe et al., 2022).

Although, in this study, we do not know the severity of patient symptoms, there is evidence of higher mental health disorders in the contexts of socioeconomic deprivation. This fact could explain why individuals with a lower SEP were more likely to be prescribed a pharmacological treatment, both from male and female GPs, than wealthier patients, and it was likely to be of greater intensity. This finding shows that people from disadvantaged backgrounds are more likely to be medicated at the first instance and hints at a possible differential treatment of this social group for the management of major depression (Giebel et al., 2020). In terms of age, elderly patients had a higher probability of being prescribed an antidepressant, although their pharmacological treatment was significantly less intense than that of younger patients. These results corroborate the fact that the prevalence of major depression increases with age and also that older people tend to present a greater risk of adverse events due to multiple comorbidities and, in the case of polypharmacy, of drug interactions (Kok and Reynolds, 2017).

Evidence of implicit gender bias in medical practice is increasing (Champagne-Langabeer and Hedges, 2021), especially in the management of depression (Cabezas-Rodríguez et al., 2020). Our results are consistent with those of other studies that draw attention to the phenomenon of medicalization as a factor in the inequalities of depression diagnosis and treatment (Ussher, 2010; Cabezas-Rodríguez et al., 2020). Other studies also revealed that female patients receive antidepressants and antipsychotic drugs more frequently than male patients, even adjusting for illness (Hohmann, 1989; Weyerer and Dilling, 1991; Alonso et al., 2004; Loikas et al., 2013).

Furthermore, our study determines that GPs’ sex does not explain the differences observed in decision-making processes regarding depression, but health-related beliefs and societal pressures have a significant impact (Berner et al., 2021). There is an unconscious bias against female patients in which their expression of their symptomatology is often perceived as an exaggeration, and knowledge of female health is insufficient (Merone et al., 2021). In this way, if the attitudes of GPs toward patients are biased from the very beginning, it will be difficult to objectively treat major depression in response to the biological and social characteristics of patients.

This study has some limitations. Due to its retrospective design, we were unable to gather qualitative information regarding the severity of major depression diagnoses or the potential non-pharmacological treatments that GPs might have suggested to their patients, and we could not assess medical referrals to mental health services. The gender identity variable was unavailable, preventing us from assessing its impact on depression management. Additionally, our analysis solely focused on the influence of GP and patient sex on the therapeutic strategy, without the ability to account for potential biases, such as the possibility of over- or under-diagnosis of major depression.

The study also has several strengths. First, the analysis used data from robust and validated administrative registers that made it possible to link the sex of the GP and patient. Furthermore, this research breaks new ground in the detection of medical gender bias in depression management, especially in the prescription of drugs, with real individual population-level data. The study results are likely to contribute to the improvement of health records and surveillance systems.

In conclusion, the GP’s sex does not seem to be an explicative factor in the differences observed in the prescription of antidepressant medication in major depression since both male and female GPs are influenced by a common social structure, biases, and stereotypes deriving from the same androcentric social and cultural contexts. Health professionals must receive a multidimensional education that allows them to address major depression individually according to the patient’s biological, psychological, and social characteristics and accompany its management with more preventive strategies and interventions to promote mental health. In addition, further investigation is needed into the behaviors of GPs toward patients in order to detect and minimize inequalities in mental health and to incorporate the gender and intersectional perspective in all areas of the healthcare system.

## Data Availability

The original contributions presented in the study are included in the article/Supplementary Material; further inquiries can be directed to the corresponding author.

## Abbreviations

ATC, Anatomical Therapeutic Chemical; CEIm, Ethics Committee for Research with Medicines (Comitè d’Ètica de la Investigació amb medicaments); CERCA, Catalan Research Centers Institution (Centres de Recerca de Catalunya); CI, confidence interval; CMBD-AP, minimum basic dataset of primary care (conjunt mínim bàsic de dades d’atenció primària); DDD, defined daily dose; ESCA, Health Survey of Catalonia (Enquesta de salut de Catalunya); GMA, adjusted morbidity groups (Grups de morbiditat ajustats); GP, general practitioner; HR, hazards ratio; ICD, International Classification of Diseases; IDIAP Jordi Gol, Foundation University Institute of Primary Healthcare Research Jordi Gol i Gurina (Fundació Institut Universitari per a la recerca a l’Atenció Primària de Salut Jordi Gol i Gurina); OR, odds ratio; PADRIS, data analytics program for research and innovation in health (Programa d’analítica de dades per a la recerca i la innovació en salut); RCA, registry of insured persons (Registre central d’assegurats); SEP, socioeconomic position; SIRE, electronic prescription database (sistema d’informació de la recepta electrònica); SISCAT, integrated public health system of Catalonia (sistema sanitari integral d’utilització pública de Catalunya).
