# Supplementary material for: The influence of general practitioner and patient sex on the treatment of major depression
Source: Front Pharmacol. 2023 Nov 9;14:1274774. doi: 10.3389/fphar.2023.1274774 (PMC10665506; doi:10.3389/fphar.2023.1274774)
Supplement: Supplementary file 1 [file Table1.DOCX]

**SUPPLEMENTARY MATERIAL**

**Supplementary Table 1.** Conversion table of antidepressants to equivalent of 40 mg fluoxetine*

*Abbreviations:* Anatomical Therapeutic Chemical (ATC); defined daily dose (DDD)
